# Four different models for simulation-based training of bronchoscopic procedures

**DOI:** 10.1186/s12890-024-02846-9

**Published:** 2024-01-09

**Authors:** Sissel Højsted Kronborg, Dan Stieper Karbing, Arman Arshad, Anna Charlotte Lundgaard

**Affiliations:** 1 Global Clinical Affairs, Clinical Application, Ambu A/S, Baltorpbakken 13, Ballerup, 2750 Denmark; 2 Department of Health Science and Technology, Aalborg University, Selma Lagerløfs Vej 249, Gistrup, 9260 Denmark; 3 Department of Respiratory Medicine, Odense University Hospital, J. B. Winsløvs Vej 4, Odense, Denmark

**Keywords:** Flexible bronchoscopy, Simulation, Model, Training, Bronchoscopy education

## Abstract

**Background:**

Flexible bronchoscopy procedures require detailed anatomical knowledge and advanced technical skills. Simulation-based training offers a patient-safe training environment that can be more efficient than patient-based training. Physical models are cheaper than virtual reality simulators and allow trainees to be acquainted with the equipment used in the clinic. The choice of a physical model for training depends on the local context. The aim of this study was to compare four different bronchoscopy models for flexible bronchoscopy training.

**Methods:**

The BronchoBoy manikin, the Koken manikin, a human cadaver, and a preserved porcine lung were included in the study. Seven physicians experienced in bronchoscopy performed a bronchoscopic airway inspection, bronchoalveolar lavage (BAL), and tissue sampling on all four models with performance evaluated by observation and participant evaluation of models by questionnaire.

**Results:**

Nineteen segments were identified in all human anatomy models, and the only significant difference found was that only the Thiel embedded cadaver allowed all participants to enter RB1 with an instrument in the working channel (*p* = 0.001). The Thiel embedded cadaver and the BronchoBoy manikin had low fluid return on BAL (22 and 52 ml), whereas the Koken manikin and the preserved porcine lung had high return (132 and 134 ml), (*p* = 0.017). Tissue samplings were only completed in the preserved porcine lung and the Thiel embedded cadaver (*p* < 0.001).

**Conclusions:**

An anatomically correct bronchoscopy is best simulated with the Koken manikin or the Thiel embedded cadaver. Bronchoalveolar lavage should be simulated with the Koken manikin or the preserved porcine lung. Tissue sampling procedures are best simulated using the Thiel embedded cadaver or the preserved porcine lung.

## Background

Flexible bronchoscopy is the standard of care for diagnostic and therapeutic procedures in the endoscopy suite, operating room, and in the intensive care unit for bedside bronchoscopy [1, 2]. Detailed anatomical knowledge and advanced technical skills are necessary to perform a successful bronchoscopic procedure [3]. Learning how to perform a flexible bronchoscopy is essential for trainees in pulmonology, thoracic surgery, ear-nose-throat surgery, and anesthesia [4, 5]. 

The traditional flexible bronchoscopy training is done using the apprenticeship model where trainees practice bronchoscopic procedures on patients under supervision. However, this method has been shown to increase the rate of patient complications, the amount of sedation, and the procedure time [6]. 

Simulation-based training offers a patient-safe training environment and has been shown to be more efficient than patient-based training [7]. There are many different modalities available for simulation-based training in bronchoscopy, including virtual reality simulators, low-fidelity models, animal models, manikin models, and human cadavers [8]. 

These modalities all have pros and cons regarding fidelity, efficacy, availability, costs, and ethical issues regarding the use of live animals or human cadavers. The virtual-reality simulators have been the focus of several scientific studies but the cost of these is a prohibitive factor for wide-spread usage [9, 10]. Physical models are considerably cheaper to acquire and allow the trainees to be acquainted with the actual flexible bronchoscopy equipment used in the clinic [10]. However, before a department or a simulation center establishes a training program based on a physical model, it must be considered which manikin, animal specimen, or human cadaver is best suited for the local context. This choice should be based on available evidence, however, only a few studies have compared these different modalities in a systematic and standardized fashion [11, 12], and no study have compared plastic manikins with a human cadaver and an animal specimen for simulation of bronchoscopic procedures. Hence, the aim of this study was to compare and discuss four different models for flexible bronchoscopy training.

## Methods

Test sessions took place in December 2021 at the Surgical Skills Centre, Ninewells Hospital and Medical School in Dundee, Scotland. In total, four different models for simulation of bronchoscopy were included (Table 1):

**Table 1:** Model characteristics

Bronchoscopy model	Model I BronchoBoy manikin	Model II Koken manikin	Model III Human cadaver	Model IV Preserved porcine lung
Version	BronchoBoy manikin (Nakhosteen, Bronchoscopy Model “SCOPIN”, Nakhosteen CLA, Coburg, Germany)	Koken manikin Bronchoscopy Training Model (Koken Bronchoscopy Training Model, KOKEN CO., LTD, Tokyo, Japan)	Thiel embedded cadaver, (Surgical Skills Centre, Ninewells Hospital and Medical School in Dundee, Scotland)	Nasco-Guard (Nasco Inflatable Healthy Swine Lung, LS03765)
Price	3734 USD [29]	7181 USD [30]	Approx. 1000 USD per use day [28]	215 USD [31]
Material	Rubber	Silicone rubber	Human tissue, Thiel embedded	Porcine lung preserved in an aqueous solution of 25% propylene glycol
Number of bronchial generations	4	5	20+ [22]	20+ [20]
Secretions	No	No	Yes	Yes

The BronchoBoy manikin (Nakhosteen, Bronchoscopy Model “SCOPIN”, Nakhosteen CLA, Coburg, Germany), which was used without further modifications.

The Koken manikin Bronchoscopy Training Model (Koken Bronchoscopy Training Model, KOKEN CO., LTD, Tokyo, Japan). This manikin has open bronchial ends which were equipped with a Luer Lock adapter (Merit Medical Systems, Inc., USA) with a balloon attached, making it possible to simulate a bronchoalveolar lavage (BAL).

A Thiel embedded cadaver (Centre for Anatomy and Human Identification, University of Dundee, United Kingdom). The cadaver was given as a voluntary donation to the Centre for Anatomy and Human Identification. After arrival at the Centre, two fluids were infused simultaneously into one artery and one vein. Hereafter, the cadaver was stored in a tank with embalming fluid for approximately 6 months [13]. The cadaver had been preserved for nearly three years at the time of the test.

**Fig. 1:**
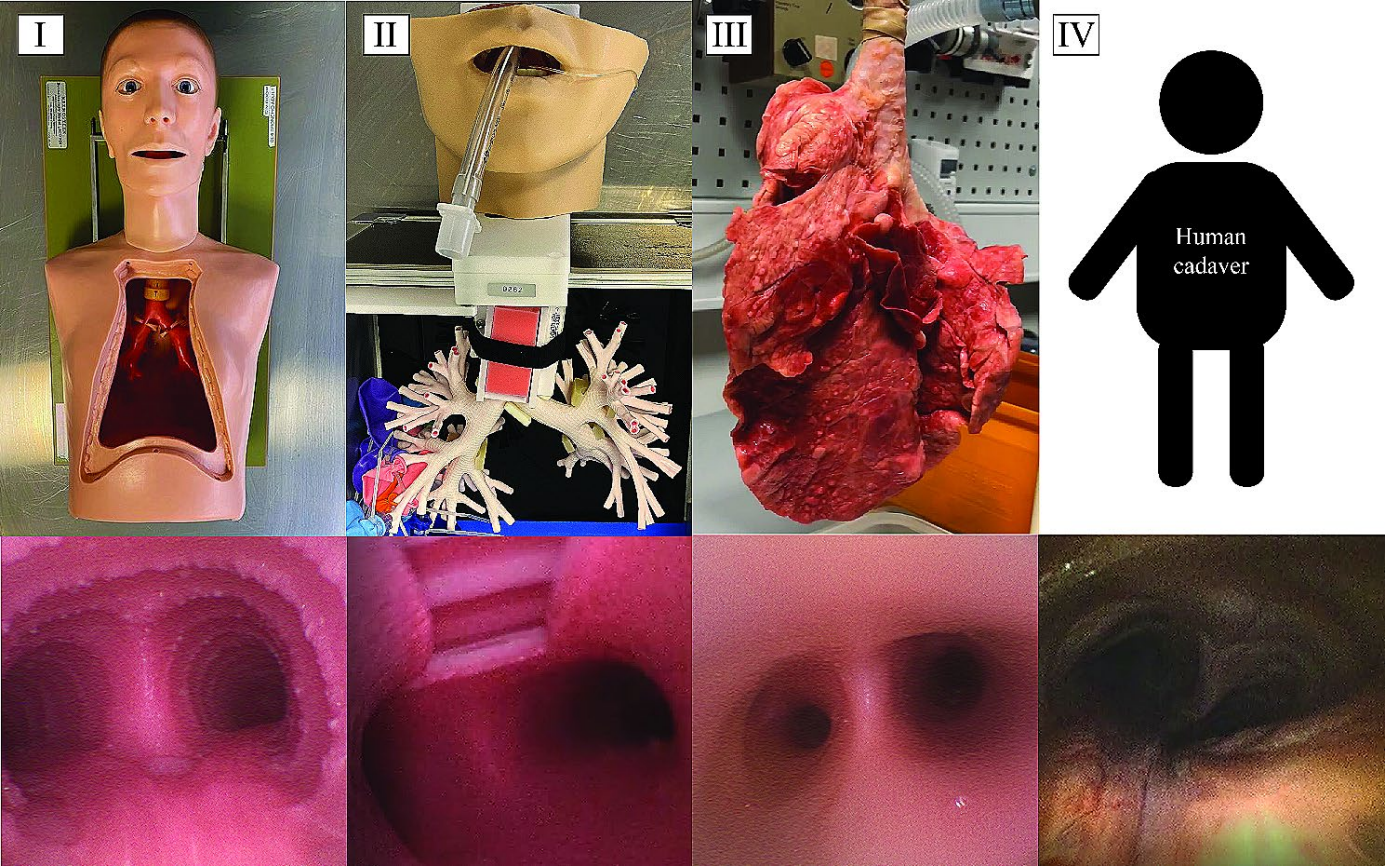
Visual appearance of the four models from outside and in trachea. (**I**) BronchoBoy manikin, (**II**) Koken manikin, (**III**) preserved porcine lung, (**IV**) human cadaver

A preserved porcine lung (Nasco-Guard, Preserved BioQuest® Inflatable Lung Kit, USA). Some similarities exist between the porcine anatomy and the human anatomy, however, as differences in the anatomy of the bronchial tree are known, the porcine lung was not included in comparison of a bronchoscopic airway inspection in this study. The visual appearance of the four models can be found in Fig. 1.

The participants were physicians from the United Kingdom with more than 2 years of experience in lower airway bronchoscopy. One individual two-hour hands-on test session was scheduled per participant. All participants started the test session by receiving a short, standardized introduction. Each test session comprised two rounds of testing with the Ambu aScope 4 Broncho Slim or Large (Ambu A/S, Denmark). For each test round, all participants performed three tasks in all four models:

1. Simulation of a bronchoscopic airway inspection, identifying all 19 segments of the bronchial tree in the BronchoBoy manikin, the Koken manikin, and the human cadaver. The participants were asked to maneuver the bronchoscope into segment 1 of the right upper lobe (RB1) and segment 6 of the right lower lobe (RB6) with and without an instrument inserted in the working channel.
2. Simulation of a BAL in all four models, advancing the bronchoscope into a chosen subsegment until wedged. In total, three times 50 milliliter were installed, followed by retrieval of as much fluid as possible using the Ambu aScope BronchoSampler (Ambu A/S, Denmark).
3. Simulation of a tissue sampling procedure performed in a random segment of the left lower lobe in all four models, using Olympus endoJaw FB-211D Disposable Biopsy Forceps (Olympus Medical, Hamburg, Germany). The sampling procedure was repeated three times for each participant.

Two assessors from Ambu A/S with training in bronchoscopy procedures and experience with the models, observed the test sessions and completed a checklist with 13 observations for each model (Table 2). The observation checklist was created by the assessors guided by an expert in flexible bronchoscopy (AA) and the clinical guidelines for bronchoscopic procedures from the British Thoracic Society [14]. A score of 1 was given if the observation was obtained in the model, and a score of 0 was given if the observation was not obtained in the model. An overview of all observations, including a definition of pass-criteria for each observation, can be seen in Table 2. No pass-criteria were defined for observations regarding advancement of the bronchoscope and secretion within the model and these observations were not given any score.

**Table 2:** Observation checklist

Task	Description	Observation	Outcome
1. Simulation of bronchoscopic airway inspection	Visualization of bronchial segments	How many segments were identified in the model?	Number of segments
		Was it possible to maneuver the bronchoscope into segment RB1 of the model without an endoscopic instrument in the working channel?	0 = RB1 not visited 1 = RB1 visited
		Was it possible to maneuver the bronchoscope into segment RB6 of the model without an endoscopic instrument in the working channel?	0 = RB6 not visited 1 = RB6 visited
	Maneuverability of bronchoscope with instruments	Was it possible to maneuver the bronchoscope into RB1 with an endoscopic instrument visible on the camera image?	0 = RB1 not visited 1 = RB1 visited
		Was it possible to maneuver the bronchoscope into RB6 with an endoscopic instrument visible on the camera image?	0 = RB6 not visited 1 = RB6 visited
	Advancement of bronchoscope	How far could the bronchoscope be advanced into a segment of the right lower lobe of the model?	Advancement of scope in cm
		How far could the bronchoscope be advanced into a segment of the left upper lobe of the model?	Advancement of scope in cm
	Secretions in the bronchial tree	How many times was it necessary to clean the lens inside the model?	Number of times
		How many times was it necessary to remove the bronchoscope to clean the lens?	Number of times
2. Simulation of BAL procedure	Installation of fluids	How much fluid was instilled in the model?	Volume in ml
		Was there any leakage of fluids from the model during the installation?	0 = Leakage of fluid at any time 1 = No leakage of fluid
	Suctioning of fluids	How much fluid was retracted from the model during suctioning?	0 = Less than 30% of instilled fluid retracted 1 = 30% or more of instilled fluid retracted
3. Simulation of tissue sampling	Sampling procedure	Was it possible to perform three sampling procedures in the model?	0 = Not possible 1 = Sampling tissue was removed x 3

After testing a model, the participants answered a questionnaire regarding its use for simulation of a bronchoscopic airway inspection, a BAL procedure, and a tissue sampling procedure as well as their perception regarding friction, color, surface structure, and maneuverability. A five-point Likert-scale from totally disagree to totally agree was used (1 = totally disagree to 5 = totally agree). An overview of all participant questions can be seen in Table 5.

All statistics were performed in SPSS (IBM SPSS Statistics for Macintosh, Version 27.0) with a 5% significance level. As data was shown to be non-normally distributed, observations are reported as median (range); questionnaire Likert scales are reported as median (interquartile range); Friedman’s tests were used to investigate any differences between models; and a post hoc analysis with Wilcoxon signed-rank tests with Bonferroni correction was used to explore significant differences. Observations regarding advancement of the bronchoscope and secretion within the model were excluded from the Friedman’s test and reported descriptively as these observations are not criteria for performance of a bronchoscopic airway inspection.

## Results

A total of 7 participants completed the test session. An overview of participant demographics is found in Table 3.

**Table 3:** Participant demographics

Participant no.	Age (years)	Sex	Position	Years of experience with bronchoscopy	Number of bronchoscopies performed weekly	Number of bronchoscopies performed in total during career
1	38	Male	Thoracic surgeon	6–9	4–10	500–999
2	36	Male	Pulmonologist	2–5	2–5	400–449
3	34	Female	Thoracic surgeon	6–9	2–5	200–499
4	53	Male	Pulmonologist & Intensivist	> 20	4–10	> 2000
5	55	Male	Thoracic surgeon	> 20	2–5	500–999
6	44	Male	Pulmonologist	16–19	4–10	1000–1999
7	47	Male	Pulmonologist	10–14	4–10	> 2000

A significant difference (*p* = 0.001) was found between the BronchoBoy manikin, the Koken manikin, and the human cadaver for a bronchoscopic airway inspection with visualization of all 19 segments and ability to enter Rb1 and Rb6 with and without an instrument in the working channel (Table 4).

**Table 4:** Observations

Observation	BronchoBoy Median (range) or actual number	Koken Median (range) or actual number	Human Cadaver Median (range) or actual number	Porcine Lung Median (range) or actual number	P (Friedman’s test)
**Task 1: Simulation of a bronchoscopic airway inspection**					
Number of segments identified	19 (19–19)	19 (19–19)	19 (19–19)	N/A	**0.001**
Number of participants entering segment RB1 without an endoscopic instrument in the working channel?	2/7	0/7	7/7	N/A	
Number of participants entering segment RB6 of the model without an endoscopic instrument in the working channel?	7/7	7/7	7/7	N/A	
Was it possible to maneuver the bronchoscope into RB1 with an endoscopic instrument visible on the camera image?	0/7	0/7	7/7	N/A	
Was it possible to maneuver the bronchoscope into RB6 with an endoscopic instrument visible on the camera image?	7/7	7/7	7/7	N/A	
How far could the bronchoscope be advanced into a segment of the right lower lobe of the model? (cm)	12.25 (9–15)	13 (11.5–16.5)	12.75 (9-18.5)	14.5 (10.5–19)	N/A
How far could the bronchoscope be advanced into a segment of the left upper lobe of the model? (cm)	8.75 (6–11)	9.5 (8-12.5)	11 (8–18)	8.75 (5–14)	N/A
How many times was it necessary to clean the lens inside the model?	0 (0–3)	0 (0–0)	0 (0–2)	1 (0–5)	N/A
How many times was it necessary to remove the bronchoscope to clean the lens?	0 (0–3)	0 (0–0)	0 (0–1)	0 (0–4)	N/A
**Task 2: Simulation of a BAL**					
How much fluid was instilled in the model? (ml)	150 (150–150)	150 (150–150)	150 (150–150)	150 (150–150)	**0.017**
Was there any leakage of fluids from the model during the installation?	6/6	2/6	2/6	0/6	
How much fluid was retracted from the model during suctioning? (ml)	52 (30–65)	132.5 (88–144)	22 (7.5–90)	134 (120–150)	
**Task 3: Simulation of a tissue sampling**					
Was it possible to perform three sampling procedures in the model?	0/7	0/7	7/7	7/7	**< 0.001**

RB1 was accessed by more participants with and without an instrument in the working channel in the human cadaver (7/7 and 7/7) than in the BronchoBoy manikin (0/7 and 2/7) (*p* = 0.033) and in the Koken manikin (0/7 and 0/7) (*p* = 0.006). All three models allowed participants to identify all bronchial segments (19(19–19)) and all participants accessed RB6 in the BronchoBoy manikin, the Koken manikin, and the human cadaver (7/7).

In the participant evaluation, a significant difference (*p* = 0.05) was found among the BronchoBoy manikin (4(3–5)), the Koken manikin (4(4–5)), and the human cadaver (5(5–5)) regarding use of the models for simulation of a bronchoscopic airway inspection (Table 5). A significant difference (*p* = 0.01) in the perceived realism of friction in the models compared to human anatomy was found, where the porcine lung (4(3–5)) was rated significantly higher than the BronchoBoy manikin (3(2–3)) (*p* = 0.02). Further, statistically significant differences were found among the models regarding color (*p* = 0.01) and surface structure (*p* = 0.03). One participant did not answer the endpoint regarding surface structure, therefore data from 6/7 participants was included only (Table 5).

**Table 5:** Participant evaluation

Endpoints	BronchoBoy Median (IQR)	Koken Median (IQR)	Cadaver Median (IQR)	Porcine lung Median (IQR)	n	P (Friedman’s test)
This model is acceptable for simulation of a bronchoscopic airway inspection.	4 (3–5)	4 (4–5)	5 (5–5)	4 (3–5)	7	**0.05**
The friction of the model was realistic compared to the human anatomy.	3 (2–3)	4 (2–4)	5 (4–5)	4 (3–5)	7	**0.01**
The color of the bronchial tree was realistic compared to the color of the human bronchial tree.	4 (3–4)	3 (2–4)	4 (4–5)	4 (3–5)	7	**0.01**
The surface structure of the bronchial tree in this model is acceptable for simulation purposes.	4 (3–4)	4 (4–5)	4 (4–5)	4 (3–4)	6	**0.03**
This model is acceptable for simulation of installation and suctioning of fluids	4 (3–4)	4 (4–5)	5 (4–5)	5 (4–5)	7	0.08
The maneuverability of the bronchoscope with and without tools was acceptable in this model.	4 (4–5)	5 (4–5)	5 (4–5)	5 (4–5)	7	0.70
This model is acceptable for simulation of a tissue sampling procedure.	3 (2–4)	4 (3–5)	5 (5–5)	5 (4–5)	7	**0.004**

In the statistical analysis of simulation of a BAL procedure data from 6/7 participants was included, as one participant did not follow the protocol in this task. A significant difference (*p* = 0.017) was found among the four models, but no significant individual differences were found in the post hoc pairwise comparisons.

A difference in the ability to simulate a tissue sampling procedure was found among the four models (*p* < 0.001) (Table 4). More participants performed three sampling procedures in the human cadaver (7/7) than in the BronchoBoy manikin (0/7) (*p* = 0.023) and in the Koken manikin (0/7) (*p* = 0.023), and more participants performed three sampling procedures in the porcine lung (7/7) than in the BronchoBoy manikin (0/7) (*p* = 0.023) and in the Koken manikin (0/7) (*p* = 0.023). Further, a significant difference (*p* = 0.004) was found among the models in acceptability for simulation of a tissue sampling procedure in the participant evaluation (Table 5), where the BronchoBoy manikin (3(2–4)) was given significant lower scores than the human cadaver (5(5–5)) (*p* = 0.03) and the porcine lung (5(4–5)) (*p* = 0.03). An overview of the most frequent participant comments can be found in Table 6.

**Table 6:** Participant comments

Model	Participant comments
Model I: BronchoBoy manikin	Bronchoscopic airway inspection: “The anatomy is good, but too easy to navigate”; “It can be used for basic training, but it cannot be used for evaluations”. BAL: “Not room enough for fluids”. Biopsy: “This is a plastic model… It is not good for biopsy as one cannot grab the tissue”. When asked about the friction, color, and surface structure of the model: “The friction is ok, but it is a bit too stiff”. “It feels sticky, but similar to the Koken manikin”; “The mucosa does not look like human tissue”. “It is more pink, it looks artificial”.
Model II: Koken manikin	Bronchoscopic airway inspection: “It is possible to visualize the segments, but they cannot be entered”. Biopsy: “It is not possible to acquire a sample in a plastic model”; “It is ok, but there should be something to grab”; “It is not as good as the porcine lung for simulation of tissue sampling”. BAL: “It is acceptable with the balloons”. When asked about the friction, color and surface structure of the model: “It feels sticky, it does not feel like in a patient”. “Not mucosal look”.
Model III: Human cadaver	Bronchoscopic airway inspection: “Seems darker”; “Easy to navigate this model”. Biopsy: “There is no blood and no vessels. Sampling is easier without blood”. When asked to advance the bronchoscope into the lung: “It looks like a bulla”.
Model IV: Preserved porcine lung	Bronchoscopic airway inspection: “Less friction compared to plastic manikins, so it is useful for basic bronchoscopy”; “Different anatomy”; “Good for training despite the different anatomy”. When asked about the friction, color and surface structure of the model: “The human lung is more red, and you can see the vessels”.

## Discussion

This study compared four models for flexible bronchoscopy training, focusing on simulation of three procedures: a bronchoscopic airway inspection, a BAL procedure, and a tissue sampling procedure. The Koken manikin and the human cadaver were preferred for simulation of a bronchoscopic airway inspection, the Koken manikin and the porcine lung were given the highest scores for simulation of BAL procedure, and the human cadaver and the porcine lung were given the highest scores for simulation of a tissue sampling procedure.

### Anatomical fidelity

Thiel embedded cadavers and plastic manikins have shown to be suitable for simulation and training of bronchoscopic airway inspection [15, 16]. In this study, simulation of a bronchoscopic airway inspection could be performed in the BronchoBoy manikin, the Koken manikin, and the human cadaver. Both manikins represent typical bronchial anatomy, and the lack of anatomical variance is a known limitation of plastic manikins [8, 12]. The anatomy of the bronchial tree in the BronchoBoy manikin was considered ‘too easy’ to navigate by several participants. No negative comments were provided for the Koken manikin. It has been shown that despite limited fidelity in plastic manikins, they are useful for training in bronchoscopy [10], suggesting that at least the Koken manikin is useful for simulation of a bronchoscopic airway inspection. Human cadavers also have a realistic anatomy and a high fidelity, but anatomical variants can be seen and might confuse novice trainees [13]. Human cadavers have been used in training bronchoscopic procedures and were favored over manikin models for educational purposes [17, 18], which is in consistency with the findings of this study. Porcine lungs are promising models for hands-on training of bronchoscopic procedures and the histological structure of the respiratory tract is similar to humans [19–21]. However, the difference in anatomy was commented on by multiple participants. Hence, the anatomical differences between the porcine and human lungs make these models inappropriate for learning human anatomy [20, 22]. 

### Training of BAL procedures

Retrieval of fluid in a BAL procedure should be more than 30% of the instilled volume [23]. In this study, several participants failed to obtain this volume of retracted fluid in the human cadaver. This is thought to be caused by a leakage found in the lungs, resulting in a spread of fluid to the abdomen and a lower volume of retracted fluid. A single Thiel embedded cadaver can be used for several years [13]. However, aging has shown to result in changes to the soft tissue of Thiel embedded cadavers [24], which might explain the leakage in the nearly three years old cadaver used in this study. When simulating the BAL procedure in the BronchoBoy manikin, more participants failed to retrieve more than 30% of the instilled amount of fluid and leakage was observed in all test sessions. In a BAL procedure, the bronchoscope must be wedged into a bronchial segment, occluding the lumen of the bronchus [14, 23]. The bronchi of the BronchoBoy manikin are closed and the lumen was found to be too wide to fully wedge the bronchoscope, resulting in a spread of fluid to the bronchial tree. Consequently, the BronchoBoy manikin is not recommended for simulation of BAL procedures. No significant difference was found between the Koken manikin and the preserved porcine lung, suggesting both models can be used for training of BAL procedures.

### Training of biopsy procedures

Tissue sampling with a biopsy forceps is an important procedure in diagnostic bronchoscopy, e.g., in cancer diagnostics [25]. Accordingly, the ability to simulate tissue sampling is essential when choosing a simulation model for training of bronchoscopy. Porcine lungs have been evaluated as a supplement to teaching of bronchoscopic techniques, including tissue sampling procedures [21]. In this study, all bronchoscopists attempted the biopsy procedure in all for models. Tissue samplings were acquired from the preserved porcine lung and the human cadaver, whereas no tissue sampling was acquired from the BronchoBoy manikin and the Koken manikin. However, this was expected due to the hard, synthetic material of the plastic manikins [26, 27]. The lack of acquiring a tissue sample from the plastic manikins was commented on by several participants, stating that this made the models less preferred for simulation of a tissue sampling procedure. Further, it was commented that the plastic manikins felt “sticky” and the appearance was artificial. By using the Thiel embedding technique, the flexibility of the tissue is preserved, making it possible to retrieve a tissue sample. Further, the tissue colors are preserved [28, 29]. In this study, the appearance of the porcine lung was commented to be too pale, but the lack of perfused blood vessels was commented on in both the porcine lung and the human cadaver. No significant difference was found between the preserved porcine lung and the human cadaver, suggesting that the differences in anatomy between porcine and human lungs are not critical for simulation of tissue sampling procedures when retrieval of a tissue sampling is possible.

### Cost & availability

Factors such as costs and availability should be considered when choosing a simulation model for bronchoscopic procedures. The price of a human cadaver is 1000 USD per use day [30]. However, a facility capable of following the regulatory requirements for cadaver studies is necessary and ethical concerns must also be considered [13, 24]. The plastic manikins can be reused over several years which makes the costs of the Koken manikin (3734 USD) and the BronchoBoy (7181 USD) reasonable one-time costs [31, 32]. Live anesthetized animals are widely used for training new doctors and could also be considered for realistic bronchoscopy training. However, there are ethical concerns when working with live animals, and the “Three Rs”: Replacement, Reduction, and Refinement should be acknowledged. Whenever possible, an alternative to animals should be used, and the minimum number of animals necessary should be used for training. Further, training should be modified in such way that any distress and pain exposed to the animal is minimized [33, 34]. These ethical concerns are overcome when using preserved porcine lungs, as the lungs are waste products from food production and can be reused for multiple procedures if stored correctly [35]. The price of a preserved porcine lung is 251 USD [36]. 

### Strengths and limitations

This is the first study to compare plastic manikins with a human cadaver and a preserved porcine lung for simulation of bronchoscopic procedures. All participants were experienced physicians, and they all evaluated all four bronchoscopy models in a fully crossed design. However, the sample size was relatively small, and it would be interesting to test the models using more bronchoscopists with different levels of experience.

It has been shown that learning benefits for novices rank higher than for more experienced physicians [37]. All participants in this study were physicians with many years of experience with real patients which might make their evaluation of physical models more critical compared to novices.

The Thiel embedded cadaver used in this study had been embedded for almost three years, which might have caused some damage to the soft tissue. This might have been avoided using a more fresh cadaver, as aging has shown to result in changes to the soft tissue of Thiel embedded cadavers [24]. In spite of this, by using a Thiel embedded cadaver, a low formaldehyde concentration is used, the lungs can be ventilated, and the flexibility of the tissue is preserved [29]. 

This study was completed using Ambu aScope 4 Broncho only. The findings of this study may be different with other single-use bronchoscopes or with reusable bronchoscopes.

### Future perspectives

Virtual reality simulators can simulate various bronchoscopy procedures and pathologies. However, the practical experience with different procedures, such as instillation of fluids and retrieval of tissue samplings, is missing. Live anesthetized porcines are often used to simulate human physiology in the evaluation of drug performance and surgical procedures [38, 39]. In this study, the preserved porcine lung was shown to be realistic for simulation of BAL procedures and tissue sampling. However, live porcines are necessary if the specific training needs include physiological reactions like ventilation, movement, and bleeding [40]. 

The included models were found to be useful for training purposes by experienced physicians, therefore the findings in this study can give an indication of which model would benefit the learning of novices in more clinical aspects. As an example, basic bronchoscopic navigation could be practiced in an anatomically correct plastic model whereas biopsy procedures would optimally require a porcine lung model or even a human cadaver. Optimally, randomized trials with solid outcome measures (e.g., generated by artificial intelligence) should be conducted to test the efficacy of the simulation-based training programs [41].

## Conclusions

No model performed best in all aspects of simulation. Simulation-based training of a complete and anatomical correct bronchoscopy should be performed using the Koken manikin or a human cadaver. Simulation of BAL procedures are optimally done with the Koken manikin or a porcine lung. Finally, practicing tissue sampling procedures can be done using human cadavers or porcine lungs. The findings in this study can help decision makers decide which model to use in training of bronchoscopic procedures.

## Data Availability

The datasets supporting the conclusions of this article are included within the article.

## Abbreviations

BAL: Bronchoalveolar lavage
RB1: Segment 1 of the right upper lobe
RB6: Segment 6 of the right lower lobe
